# Immunomodulatory effect of extracellular vesicles from *Entamoeba histolytica* trophozoites: Regulation of NETs and respiratory burst during confrontation with human neutrophils

**DOI:** 10.3389/fcimb.2022.1018314

**Published:** 2022-10-28

**Authors:** César Díaz-Godínez, Diana G. Ríos-Valencia, Samuel García-Aguirre, Santiago Martínez-Calvillo, Julio César Carrero

**Affiliations:** ^1^ Departamento de Inmunología, Instituto de Investigaciones Biomédicas, Universidad Nacional Autónoma de México (UNAM), Ciudad de México, Mexico; ^2^ Unidad de Biomedicina, Facultad de Estudios Superiores Iztacala, Universidad Nacional Autónoma de México, Tlalnepantla, EM, Mexico

**Keywords:** *Entamoeba histolytica*, human neutrophil, extracellular vesicles (EVs), NETs, ROS, immunomodulation, proteomic analysis

## Abstract

Parasites release extracellular vesicles (EVs) which, in some cases, modulate the host’s immune response contributing to the establishment of the infection. In this work we have isolated and characterized the EVs released by trophozoites of the human protozoan parasite *Entamoeba histolytica*, the causal agent of amoebiasis, when alone or in coculture with human neutrophils, and determined their effect on neutrophil NETs and ROS production. Nanoparticle tracking analysis showed that amoebic EVs are variable in size, ranging from less than 50 nm to nearly 600 nm in diameter (average of 167 nm), whereas neutrophil EVs are more uniform in size, with an average of 136 nm. In cocultures amoeba:neutrophil (1:100) most EVs are 98 nm in size, which is the typical size of exosomes. EVs from amoebae and neutrophils showed almost equal levels of ROS, which were considerably increased in EVs from cocultures. Uptake of amoebic EVs by neutrophils was demonstrated by fluorescence and resulted in a significant reduction in the oxidative burst and NET release triggered by PMA, ionophore A23187, or the amoebae itself used as stimuli. Interestingly, uptake of EVs from cocultures did not affect ROS production, but instead caused a greater delay in the onset of NETs release and in their quantity. A comparative proteomic analysis between the EVs of amoebae and neutrophils separately vs the cocultures showed a similar distribution of protein categories in the GO analysis, but differences in the expression and abundance of proteins such as the N-acetyl-D-galactosamine (GalNAc) inhibitable surface lectin and calreticulin in amoeba EVs, and various antimicrobial molecules in neutrophil EVs, such as lactoferrin and myeloperoxidase. These results highlight the importance of EVs in the immunomodulatory effects exerted by amoeba on human neutrophils.

## Introduction

Extracellular vesicles (EVs) represent a heterogeneous group of membranous bodies produced by practically all living organisms, from bacteria to humans (Bose et al., 2021; Simon et al., 2018; Rizzo et al., 2020; Liu et al., 2021). Currently, the existence of three types of EVs is accepted according to their characteristics of size, cargo, and secretion route: exosomes (30-150 nm), microvesicles (100 up to 1 μm) and apoptotic bodies (500 nm up to >5 μm) (Doyle and Wang, 2019). The content of the EVs includes a wide diversity of proteins, different types of RNA (mRNA, tRNA, siRNA, lncRNAs), DNA, and even complete organelles (Doyle and Wang, 2019; Veziroglu and Mias, 2020; Yokoi and Ochiya, 2021). In addition, they possess surface molecules that also provide them functional properties (Burrello et al., 2021). EVs are critical elements of cell-to-cell communication since they mediate complex functions such as cell growth and proliferation, differentiation, response to stimuli, immunomodulation, and even pathological processes (Veerman et al., 2019; Zhao et al., 2021; Hanayama, 2021).

The host-parasite interaction is a complex phenomenon that involves, on the one hand, the generation of an immune response by the host to eliminate the invader, and on the other, the manipulation or evasion of that response by the parasite to survive (Cheeseman and Weitzman, 2015; Acharya et al., 2017; Cuesta-Astroz et al., 2019). Studies suggest that EVs play a critical role in this interplay. Thus, it has been shown that the EVs released by some parasites can suppress the host’s immune response, changing the phenotype of different leukocytes by suppressing responses such as chemotaxis, phagocytosis, or respiratory burst (Castelli et al., 2019; Soto -Serna et al., 2020; Díaz-Godínez et al., 2022). On the other hand, EVs released by host leukocytes usually have baseline antimicrobial activity that changes in intensity according to the degree of threat posed by the pathogen (Lorincz et al., 2015; Shopova et al., 2020).

*Entamoeba histolytica* is the protozoan parasite responsible for amebiasis, a highly prevalent disease in developing countries that is acquired by ingesting water or food contaminated with fecal matter that carry cysts. The infection causes different pathologies, from diarrhea during the invasion by the trophozoites of the intestinal epithelium, to abscesses in organs such as the liver and brain once the parasite migrates out of the intestine (Kantor et al., 2018; Carrero et al., 2020). When the amoeba invades the tissue, an inflammatory response is initiated with the recruitment of large numbers of neutrophils that in some cases seem to contribute, together with the INF-mediated Th1 response, to protection (Nakada-Tsukui and Nozaki, 2016). However, in the case of the hamster amoebic liver abscess model, early arrival of neutrophils and their lysis by the amoeba has been associated with extensive tissue destruction and pathology (Olivos-García et al., 2007). We have previously described that viable trophozoites of *E. histolytica* can induce neutrophil lysis by triggering the release of neutrophil extracellular traps (NETs), DNA structures with antimicrobial properties, but which can also contribute to the pathology of some diseases when exacerbated (Mitsios et al., 2017). To decipher the mechanism of NETosis by amoebae, we previously reported that it is partially dependent on active transfer of components between cells. Specifically, the process requires the transfer of ROS from the amoeba to the neutrophil and the transfer of myeloperoxidase (MPO) from the neutrophil to the surface of the amoeba during intimate contact between both cells (Avila et al., 2016; Díaz-Godínez et al., 2018; Díaz Godínez et al., 2021). Although the effects of this interaction are evident, the mechanism of transfer between the two cells has not been elucidated; it is tempting to speculate that it could involve EVs.

The unique previous report on EVs from amoebae showed that these structures, released by encystment-induced trophozoites of the reptilian amoeba *Entamoeba invadens*, can promote encystment of induced trophozoites, suggesting that amoebae can transfer information using their EVs (Sharma et al., 2020). However, no studies have yet been carried out to demonstrate the interaction of *E. histolytica* EVs with human immune cells and their effect on the regulation of effector mechanisms. In this work we isolated EVs released by *E. histolytica* trophozoites (EVs A), EVs from human neutrophils (EVs N) and EVs from a coculture amoebae:neutrophils (EVs N+A), determined their proteome, and evaluated their ability to induce NETosis and to affect the NETosis and respiratory burst of neutrophils under several stimuli.

## Materials and methods

### *E. histolytica* trophozoite culture

*E. histolytica* trophozoites were obtained as we described previously (Díaz-Godínez et al., 2021). In brief, *E. histolytica* trophozoites (HM1:IMSS strain) were axenically cultured in TYI-S-33 medium supplemented with Diamond vitamin tween solution (Merck) and 15% heat-inactivated adult bovine serum (Microlab). Trophozoites were grown during 72 h at 37°C until log phase and harvested by ice chilling during 5 min. Trophozoites were centrifuged at 1400 rpm during 5 min at 10°C and the pellet was resuspended in PBS pH 7.4. Amoebae were counted in hemocytometer and preserved at room temperature until use.

### Neutrophil isolation

Neutrophils were obtained from peripheral blood of healthy volunteers according to García-García et al. (2013) using Ficoll-Paque^®^ gradient (GE Healthcare) and hypertonic shock to lyse erythrocytes. Cells were resuspended in PBS pH 7.4, counted in hemocytometer and reserved at 4°C until use. This study was carried out in accordance with the recommendations and approval of the Ethical Committee for Studies on Humans of the Instituto de Investigaciones Biomédicas, UNAM (Ethical approved number: FMED/CI/RGG/013/01/2018). All subjects signed a written informed consent.

### Extracellular vesicles isolation

For EV isolation, 7.5 × 10^6^ neutrophils or 7.5 × 10^5^ trophozoites were centrifuged at 1400 rpm for 5 min and washed 3 times with PBS pH 7.4. After last washing, neutrophils or trophozoites were resuspended in 3 ml of free-serum RPMI-1640 medium (Biological Industries) and culture in 24 well-plates for 1 h at 37°C. After incubation, the culture supernatant was collected in conical tubes and centrifuged at 1400 rpm for 5 min. Free cell supernatant was obtained collecting only 2.5 ml of culture media after centrifugation and filtered through 0.22 μm. The EVs were purified using Total Exosome Isolation (from cell culture media) reagent (Invitrogen, cat 4478359) according to manufacturer instructions. EVs were resuspended in filtered PBS pH 7.4 and protein concentration was determined with Nanodrop 2000 equipment (Thermo Fisher). For EVs derived from trophozoites-neutrophil coculture, 2.5 ml of free-serum RPMI-1640 containing 7.5 ×10^6^ neutrophils were placed in 24 well-plate and immediately were added 0.5 ml of the same medium containing 7.5 × 10^5^ trophozoites (ratio 10:1; final volume of 3 ml). Coculture was incubated for 1 h at 37°C and the methodology was the same as described above.

### Nanoparticle tracking analysis

Size of EVs was determined by diluting unstained EVs in 1 ml of filtered PBS pH 7.4 and analyzed with Nanosight NS3000 (Malvern Panalytical) equipment in triplicates.

### Transmission electron microscopy

EVs were processed for TEM as previously reported (Ramírez-Ricardo et al, 2021). In brief, EVs (~ 10 μg) were suspended in 100 μl sterile PBS, from which 30 μl was adsorbed for 5 min on carbon-coated copper grids with mesh formvar (0.3%) at room temperature. The grids were stained with uranyl acetate solution (2%) for 30 s and the excess of fluid was removed. The grids were air-dried and analyzed at 80 kV using a JEM-1400 transmission electron microscope (JEOL, Ltd. Japan) equipped with a digital camera Veleta (Olympus SIS. Germany).

### Lipid and ROS staining of EVs

A volume of 10 µl EVs suspension (from neutrophils, trophozoites or coculture) were mixed with 3 µl of DiO (Thermo Fisher) fluorescent colorant (1:10 dilution in PBS pH 7.4) and incubated during 5 min at room temperature, or with 10 µM 2′,7′-dichlorofluorescein diacetate (H_2_DCFDA; Merck) and incubated for 30 min at 37°C in the dark. After this time, 1.5 µl of stained EVs were placed in 1.5% low melting point agarose bed (BioRad; 3 mm thickness) and covered with a coverslip. EVs were analyzed using a Nikon A1R+ confocal microscope for pictures and video. Images were amplified using NIS elements viewer software (Nikon).

### Amoebic EVs: Neutrophils interaction assays

To evaluate the interaction between amoebic EVs and human neutrophils, 10 µg of DiI (Thermo Fisher) stained EVs A (3 µl DiI, diluted 1:10 in PBS, 5 min) were added to 100 µl of RPMI-1640 medium containing 2 × 10^5^ human neutrophils. Cells were incubated during 10 min and then formaldehyde-fixed (3.5%) for 20 min. After fixation cells were washed 3 times with PBS, stained with Hoechst (5 µg/ml) and resuspended in 50 µl of Fluroshield™ (Merck). Samples were mounted and visualized using a Nikon A1R+ confocal microscope for pictures To evaluate the transfer of ROS of EVs to the neutrophils, 10 μg of H_2_DCFDA stained EVs were added to 100 µl of RPMI-1640 medium containing 2 × 10^5^ human neutrophils and incubated for 30 min at 37°C in the dark. The cells were processed and analyzed as before.

### Intracellular and EVs ROS quantitation

ROS measurement was performed as we described previously with some modifications (Díaz-Godínez et al., 2021). In brief, neutrophils (5 × 10^5^) were resuspended in 500 µL of PBS added with 10 µM 2′,7′-dichlorofluorescein diacetate (H_2_DCFDA; Merck) and incubated for 30 min at 37°C in the dark. Cells were centrifuged at 4000 rpm for 2 min and resuspended in 500 µL of RPMI-1640 supplemented with 5% FBS. Subsequently, 100 µL of suspension (1 × 10^5^ neutrophils) was transferred to 96 well plate and allowed to sediment for 10 min at 37°C. EVs A, EVs N or EVs N+A, in a quantity equivalent to 10 µg of protein, were added to the H_2_DCFDA-treated neutrophils and then immediately stimulated or not with PMA (50 nM), A23187 (10 µM) or 1 × 10^3^ *E. histolytica* trophozoites. Fluorescence intensity was measured after incubation during 1 h at 37°C from the well bottom in the spectrofluorometer Synergy HTX (Bio Tek) using 485 nm excitation and 528 nm emission filters.

For quantitation of ROS in EVs, EVs N, EVs N or EVs N+A in a quantity equivalent to 10 µg of protein were added to 200 µL of PBS with 10 µM 2′,7′-dichlorofluorescein diacetate (H_2_DCFDA; Merck) in a 96 well plate and incubated for 10 min at 37°C in the dark. Fluorescence intensity was measured after incubation during 4 h at 37°C as mentioned above. Hydrogen peroxidase diluted 1:10,000 was used as positive control.

### NET quantitation assay

NET quantitation was performed as we described before with some modifications (Díaz-Godínez et al., 2021). In brief, neutrophils (5 × 10^5^) were centrifuged at 4000 rpm for 2 min and resuspended in 500 µL of RPMI-1640 medium supplemented with 5% fetal bovine serum (FBS, Gibco) and 500 nM SYTOX^®^ Green (Invitrogen). A volume of 100 µL of cell suspension (1 × 10^5^ neutrophils) was added to a 96 well plate, allowed to sediment for 20 min at 37°C, and then treated with 10 µg of protein of EVs N, EVs A or EVs N+A. Immediately, cells were stimulated or not with PMA (50 nM), A23187 (10 µM) or 1 × 10^3^ *E. histolytica* trophozoites. The fluorescence was measured during 4 h from the well bottom using a spectrofluorometer Synergy HTX with 485 nm excitation and 528 nm emission filters.

### Mass spectrometry

EVs A, EVs N or EVs N+A, equivalent to 100 µg of protein each, were run on 12% polyacrylamide gels and run just enough time to allow the mixture of proteins to enter the stacking gel and concentrate as a coarse band. After staining with Bio-Safe Coomasie G-250 stain (BioRad), the bands were cut under sterile conditions and subjected to in-gel trypsin digestion. Briefly, gel pieces were washed with 50 mM ammonium bicarbonate (Acros) in 50% acetonitrile (Fisher), reduced with dithiothreitol (Acros) and alkylated with iodoacetamide (Sigma), washed again, and impregnated with ~75 µL of 6 ng/µL trypsin (trypsin gold; Promega) solution overnight at 37°C. The resulting peptides were extracted using solutions of 50% and 80% acetonitrile (ACN) with 0.5% formic acid (Millipore), and the recovered solution was dried down in a vacuum concentrator.

Dried peptides were dissolved in 60 µL of 0.1% trifluoroacetic acid (TFA, Sigma), and desalted using 2-core MCX stage tips (3M, 2241) (Rappsilber et al., 2003). The stage tips were activated with ACN followed by 3% ACN with 0.1% TFA. Next, samples were applied, followed by two washes with 3% ACN with 0.1% TFA, and one wash with 65% ACN with 0.1% TFA. Peptides were eluted with 75 µL of 65% ACN with 5% NH_4_OH (Sigma), and dried.

#### LC-MS methods

Samples were dissolved in 25 µL of water containing 2% ACN and 0.5% formic acid. Two µL (0.5 µg) were injected onto a pulled tip nano-LC column with 75 µm inner diameter packed to 25 cm with 3 µm, 120 Å, C18AQ particles (Dr. Maisch). The peptides were separated using a 120 min gradient from 3 – 28% ACN, followed by a 7 min ramp to 85% ACN and a 3 min hold at 85% ACN. The column was connected in line with an Orbitrap Lumos *via* a nanoelectrospray source operating at 2.2 kV. The mass spectrometer was operated in data-dependent top speed mode with a cycle time of 2.5s. MS1 scans were collected at 120,000 resolution with a maximum injection time of 50 ms. Dynamic exclusion was applied for 15 s. HCD fragmentation was used followed by MS2 scans in the ion trap with 35 ms maximum injection time.

#### Database searching and label-free quantification

The MS data was searched using SequestHT in Proteome Discoverer (version 2.4, Thermo Scientific) simultaneously against three databases: *E. histolytica* (Uniprot, containing 8171 entries, retrieved 26/8/2021), human (Uniprot, containing 20443 reviewed entries, retrieved 13/9/2019), and a list of common laboratory contaminant proteins (Thermo Scientific, 298 entries, 2015). Enzyme specificity for trypsin was set to semi-tryptic with up to 2 missed cleavages. Precursor and product ion mass tolerances were 10 ppm and 0.6 Da, respectively. Cysteine carbamidomethylation was set as a fixed modification. Methionine oxidation, protein N-terminal acetylation, methionine loss, and methionine loss plus acetylation were set as variable modifications. The output was filtered using the Percolator algorithm with strict FDR set to 0.01. Label-free quantification was performed in Proteome Discoverer with normalization set to total peptide amount. The comparison of the EVs derived from the cultures separately with those from the co-cultures was done by spectral counts. Gene ontology analysis was performed using PANTHER GO platform.

### Statistical analysis

Statistical significance was tested with U-Mann Whitney test. Data are reported as mean ± SD. A p value ≤ 0.05 was considered statistically significant. All experiments were carried out in three independent experiments.

## Results

### The *E. histolytica* trophozoites release EVs of different sizes when alone or in coculture with neutrophils

**Figure 1:**
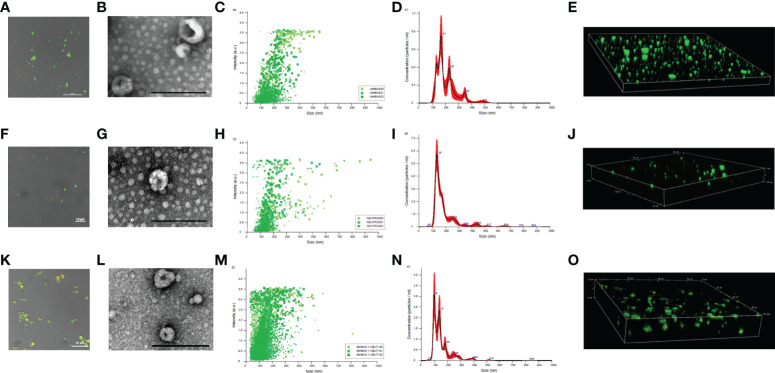
Size determination of EVs derived from *E. histolytica* trophozoites, neutrophils and coculture. EVs from amoebae **(A)**, neutrophils **(F)** and cells in coculture **(K)** were isolated from culture media and stained using DiO (1:10 dilution). EVs were place in 1.5% low melting point agarose for visualization. Scale bar sizes are indicated in each micrograph. TEM of EVs isolated from amoebae **(B)**, neutrophils **(G)** and coculture **(L)**; scale bar represents 200 nm. EVs size was determine with Nanosight NS3000 for triplicates. It is showed dot plots and histograms for EVs from amoebae **(C, D)**, neutrophils **(H, I)**, and coculture **(M, N)**, respectively. 3D reconstruction of the DiO-stained EVs from amoebae **(E)**, neutrophils **(J)** and coculture **(O)** was obtained through confocal microscope.

The pellet obtained from *E. histolytica* trophozoites cultures by using the exosome isolation kit was stained with DiO dye and observed by confocal microscopy to determine lipid membranes. As shown in **Figure 1A** were positive for lipid membranes, exhibiting green fluorescence. TEM analysis in **Figure 1B** shows that amoebic isolated nanoparticles are less than 200 nm in size and that their morphology is close-to-spherical with a central cleft, confirming that they are EVs. We also purified EVs from human neutrophils (**Figures 1F, G**) and from cocultures neutrophils: trophozoites (100:1; **Figures 1K, L** ) with similar characteristics. Nanoparticle Tracking Analysis (NTA) showed that the amoebic EVs obtained from three independent samples range in size from less than 50 nm to almost 600 nm (**Figures 1C–E**; **Supplementary Video 1**). As shown in **Figure 1D**, an enrichment of EVs with a diameter of approximately 167 nm was obtained. In addition, peaks of EVs with a diameter of 141, 230, 348 and 483 nm were also observed.

In the case of neutrophils, the EVs released into the medium were more uniform in size with an average size of 136 nm (**Figures 1H–J**; **Supplementary Video 2**). Larger EVs are observed but in very small amounts. When neutrophils were cocultured with amoebic trophozoites, EVs of smaller size than with cells in independent cultures were identified, most showing a size of 98 nm, which is typical of exosomes (**Figures 1M–O**). Also, EVs of 137 nm (like those of neutrophils alone), and 180 nm (like those of amoebae alone), were also observed (**Figure 1N**). Interestingly, confocal microscopy observations showed that EVs from the coculture tend to aggregate, suggesting the interaction between EVs N and EVs A (**Figures 1K, L** and **Supplementary Video 3**).

### Human neutrophils incorporate amoebic EVs

**Figure 2:**
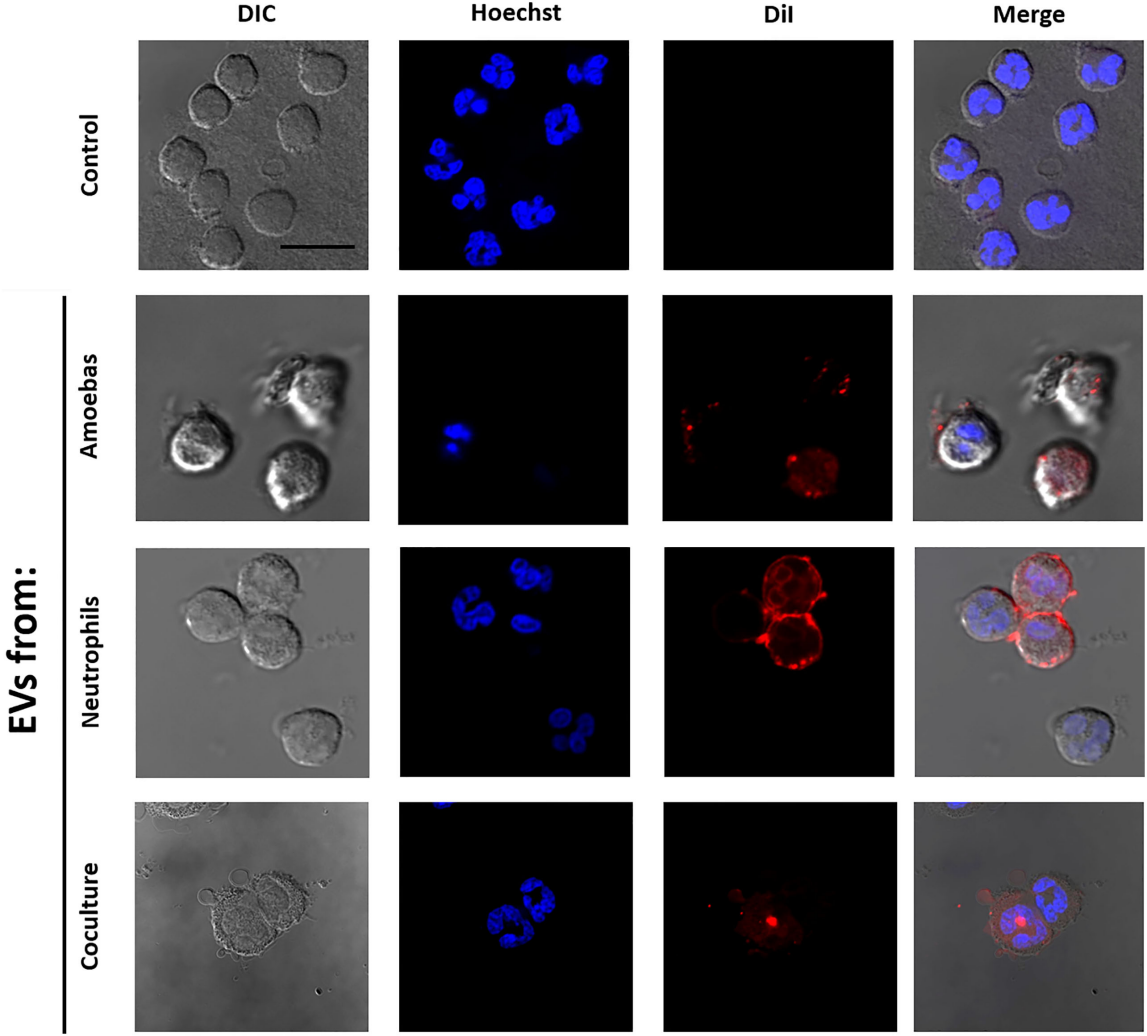
Interaction of EVs with human neutrophils. EVs were isolated from *E. histolytica* trophozoites, neutrophils or coculture; then, EVs were stained using DiI and added to neutrophil (2 x 10^5^) cultures. After 10 min of interaction, neutrophils were fixed, stained with Hoescht (5 µg/ml), and mounted for visualization using Fluorshield™. Neutrophils in the absence of exosomes were used as control. Scale bars represent 20 µm and all figures are in the same magnification.

We wondered if EVs A were internalized by human neutrophils or whether they fused with the cytoplasmic membrane. For this, EVs A, EVs N and EVs N+A were stained with DiI dye and added to neutrophils. **Figure 2** shows that neutrophils treated with the stained EVs A showed intense red fluorescence dots associated to the cell membrane and dots and diffuse red fluorescence in the cytoplasm, but no other cell compartments, including the nucleus. This suggests the fusion of EVs A with the human neutrophil membrane, but also the incorporation of complete EVs into the cytosol. Similar uptake of EVs Nand EVs N+A by human neutrophils were also observed (**Figure 2**). Control untreated neutrophils exhibited multilobed nuclei (Hoechst stained) and no red fluorescence was observed in the membrane or cytosol as expected.

### Amoebic EVs carry ROS and transfer them to the neutrophils

**Figure 3:**
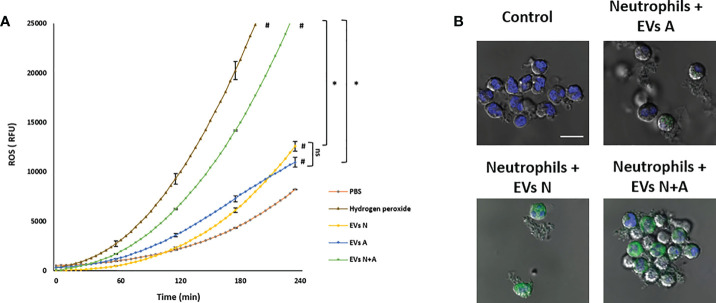
ROS detection in EVs from trophozoites, neutrophils and coculture and transfer to neutrophils. **(A)** Freshly isolated EVs (10 µg) were treated with H_2_DCFDA and fluorescence was measured during 4 h and reported as fluorescence relative units (FRU). PBS was used as background control and hydrogen peroxide as positive control. Graphs represent mean of three independent experiments and ± SD was placed every hour. # Significant difference respect to PBS control (p<0.05). *(p<0.01). **(B)** Confocal microscopy images showing the incorporation of H2CFDA-stained EVs into human neutrophils. Scale bars represent 20 µm and all figures are in the same magnification.

The treatment with H2DCFDA of EVs released by amoebae and neutrophils in independent cultures showed that they carry similar ROS activity that increases over time, being more evident from 2 h onwards of incubation (**Figure 3A**). It is noteworthy that ROS levels were more than twofold higher in EVs N+ than in EVs from independent cells, which suggests that the confrontation between the two types of cells stimulates a greater load of ROS in the EVs, or that more ROS are generated within them. As expected, no ROS were detected in PBS and high levels of ROS were found in the hydrogen peroxide positive control. **Figure 3B** shows that EVs of all samples transfer ROS to the human neutrophils as H_2_CFDA-stained EVs are seen in the cytosol of the cells. EVs N+Aseem to transfer the highest level of ROS.

### The *E. histolytica* EVs downregulate human neutrophil respiratory burst and NETosis

To determine the EVs effect on ROS production and NET formation, EVs A (10 µg) were added to neutrophils untreated or treated with different stimuli (PMA, A23187 ionophore and amoebae).

**Figure 4:**
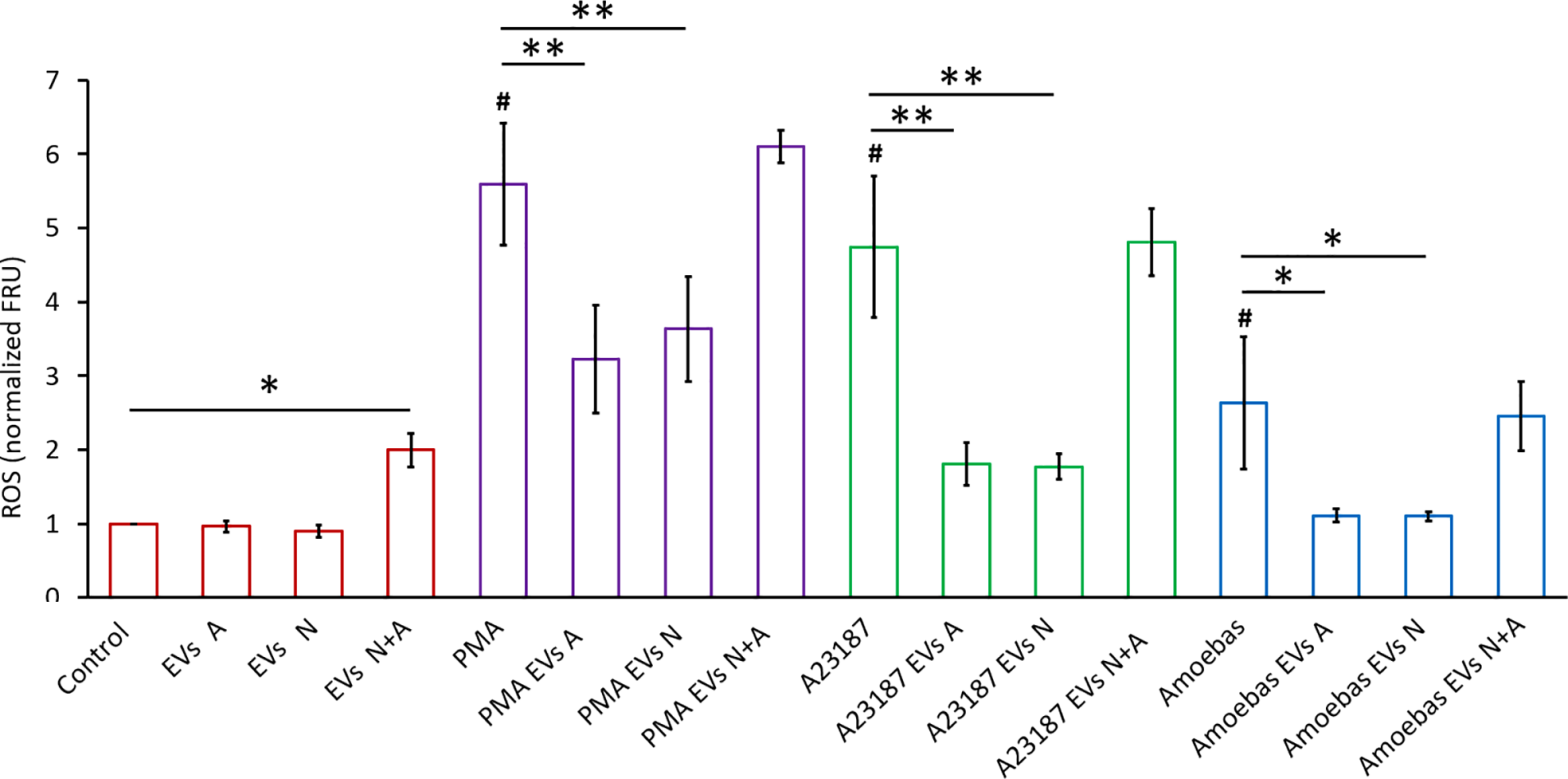
ROS detection in neutrophils stimulated with EVs. H_2_DCFDA stained neutrophils (1 × 10^5^) were transferred to 96 well plates and allowed to sediment. EVs (10 µg) from trophozoites (Exo A), neutrophils (Exo N), or coculture (Exo N+A) were added to neutrophils and then immediately stimulated or not with PMA (50 nM), A23187 (10 µM) or 10^3^ *E. histolytica* trophozoites. Fluorescence was measured after 1 h, data were normalized with respect to control and fluorescence relative units (FRU) were graphed. Graphs represent mean ± SD of three independent experiments. *p < 0.05; **p < 0.01; # significant difference respect to control.

**Figure 4** shows that unstimulated control neutrophils produce a basal amount of ROS that is not affected when EVs A or EVs N were added. However, a slight but significative increase in ROS production (p<0.05) was observed when EVs N+A were added. Treatment of neutrophils with PMA, A23187 or amoebae (ratio amoeba:neutrophil 1:100) induced an increase in ROS production, as expected (amoebae inhibits the neutrophil respiratory burst but at ratios higher than 1:50; Díaz-Godínez et al, 2021). Interestingly, when EVs A or EVs N were added to the neutrophil-stimulated cultures, they significantly reduced the oxidative burst triggered by all stimuli (p<0.01). In contrast to PMA-stimulated neutrophils, where EVs only partially reduced ROS production, the respiratory burst was drastically decreased in ionophore-stimulated neutrophils and completely inhibited in ameba-stimulated cells. Noteworthy, the inhibitory capacity of the respiratory burst was lost with the EVs N+A, since the ROS levels remained the same as in the stimulated but untreated controls (**Figure 4**).

**Figure 5:**
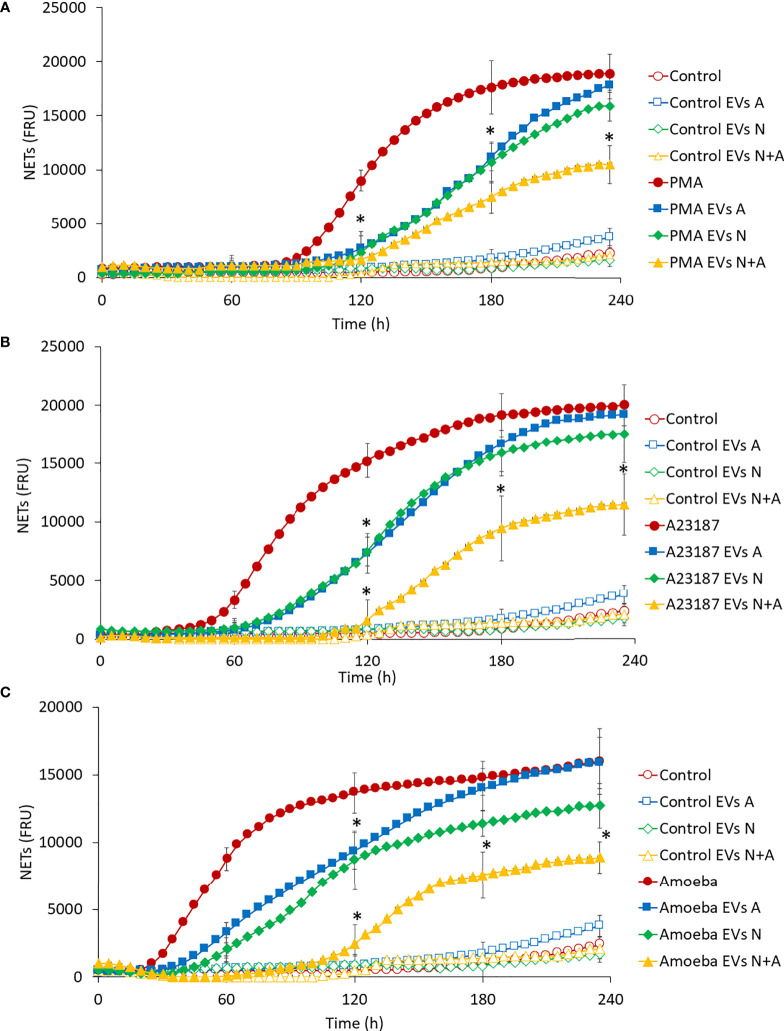
Effect of EVs on NET release in human neutrophils. Neutrophils (1 × 10^5^) were culture in RPMI-1640 medium added with SYTOX^®^ Green and treated with EVs (10 µg of protein) from trophozoites, neutrophils, or coculture. Immediately, cells were stimulated or not with PMA **(A)**, A23187 **(B)** or 10^3^ *E. histolytica* trophozoites **(C)**. Fluorescence was measured during 4 h and reported as fluorescence relative units (FRU). Graphs represent mean of three independent experiments and ± SD was placed every hour. Measured controls were the same for all graphs. *p < 0.05; significant difference respect to stimulated neutrophils in the absence of EVs.

Regarding the effect of EVs on NET release, **Figure 5** shows that EVs A induced a slight release of NETs after 180 min stimulation (Control EVs A) when compared to EVs N (Control EVs N) and those from coculture (Control EVs A+N), whose NET release levels were the same as the untreated control (Control). As expected, PMA, A23187 and amoebae induced the release of NETs at 90, 50 and 20 min after stimulation, respectively (**Figures 5A–C**). Of note, the addition of EVs A or EVsN to stimulated neutrophils caused a significant delay of 20 min in the onset time (110, 70 and 40 min) and rate of NET release. However, at 4 h post-exposure, the amount of NETs reaches, or even exceeds, the amount of NETs in stimulated neutrophils. Interestingly, EVs N+A caused a greater delay in the onset of NET release than EVs from cultures alone (from 110 to 130 min in PMA-, from 70 to 120 min in A23187- and from 40 to 110 min in amoeba-treated neutrophils) and decreased their release throughout the 4-h evaluation period (**Figures 5A–C**, respectively).

### Proteomic analysis of *E. histolytica* and neutrophil EVs

**Figure 6:**
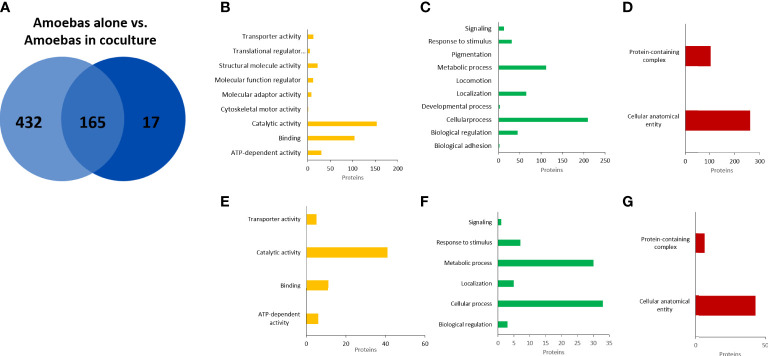
Proteomic analysis of EVs released from *E. histolytica* trophozoites. Venn diagram of comparted proteins between amoebae culture alone and amoebae cocultured with neutrophils **(A)**. Gene ontology (GO) analysis performed in PANTHER GO of molecular function **(B)**, biological processes **(C)**, and cellular components **(D)** of EVs proteins from amoebas alone. **(E–G)**, similar analysis of EVs proteins from amoebas in coculture.

EVs isolated from four cultures of *E. histolytica* trophozoites, from two human neutrophil preparations and from four cocultures neutrophil:amoebae were analyzed by mass spectrometry. A total of 597 different annotated proteins were identified from the four *E. histolytica* EV samples (**Figure 6A**). Gene ontology (GO) analysis identified the molecular function of 316 proteins, of which the catalytic activity function is the most representative (comprising 44% of them), followed by binding proteins (representing almost 30%) (**Figure 6B**). The analysis by biological processes identified 443 proteins, most of them related to cellular and metabolic processes. Interestingly, many proteins involved in biological regulation and response to stimulus were also identified (**Figure 6C**). Finally, analysis of the data based on cellular components identified 339 proteins distributed in only two categories: cellular anatomical entity and protein-containing complex (**Figure 6D**). On the other hand, in the EVs N+A, the identification of only 182 proteins of the amoeba was achieved, of which 165 are shared with the EVs A (**Figure 6A**). GO analysis showed that the distribution of these proteins by molecular function, cellular components and biological processes is similar to that of EVs A (**Figures 6E–G**). Most of the remaining 17 amoeba proteins identified only in coculture EVs correspond to enzymes, transporters, and uncharacterized proteins (**Supplementary Table 1**). Interestingly, among the 165 shared proteins, notable increases in EVs from amoebae in coculture vs EVs from amoebae alone were found in the relative abundance of proteins such as the 170-kDa subunit of GalNAc-inhibitable lectin (5.8-fold), calreticulin (5.1), amoebapore (4), glucosidase (4.6) and cysteine proteinase (3.3), among others (**Supplementary Table 2**).

**Figure 7:**
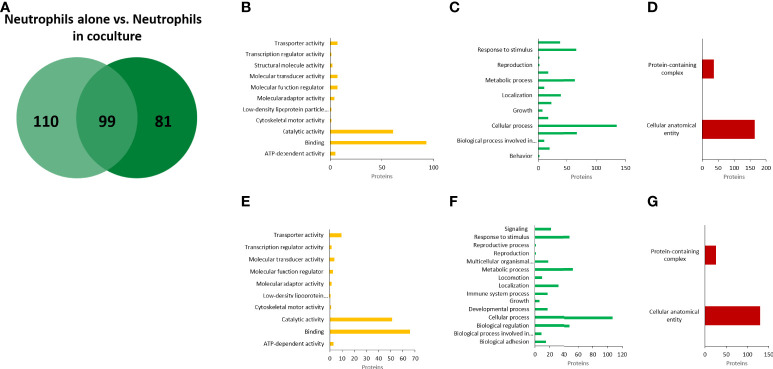
Proteomic analysis of EVs released from human neutrophils. Venn diagram of comparted proteins between neutrophils culture alone and neutrophils cocultured with amoebae **(A)**. Gene ontology (GO) analysis performed in PANTHER GO of molecular function **(B)**, biological processes **(C)**, and cellular components **(D)** of EVs proteins from neutrophils alone. **(E–G)**, similar analysis of EVs proteins from neutrophils in coculture.

In the case of neutrophils, we identified 209 annotated proteins with high confidence values (**Figure 7A**). Notably, most of them were classified in the same GO categories as the proteins from EVs A: catalytic activity and binding by molecular function; cellular and metabolic processes, biological regulation, and response to stimulus by biological processes; and cellular anatomical entity and protein-containing complex by cellular components (**Figures 7B–D**). In coculture EVs, 180 neutrophil proteins were identified, of which 99 matched to neutrophil EVs alone, and 81 were unique to neutrophil EVs in coculture (**Figures 7A, E–G**). From the 81 proteins expressed by the neutrophils only in the presence of amoeba, 7 anti-microbial proteins were identified (**Supplementary Table 3**). Noteworthy, among the 99 shared proteins, notable increases in EVs from neutrophils in coculture vs EVs from neutrophils alone were found in the relative abundance of remarkable microbicidal proteins such as neutrophil defensin 4 (26-fold), lactotransferrin (14), myeloperoxide (10), neutrophil elastase (7.5), among others (**Supplementary Table 4**).

### Exosomal markers proposed in *E. histolytica* and proteins of interest

Even when *E. histolytica* does not have the most classic marker of exosomes (i.e. tetraspanin, whose absence in EVs was previously reported in Sharma et al. (2020) and confirmed by us in this work), here we identified a series of 16 EV proteins (**Table 1**) listed among the 100 proteins most frequently identified in exosomes, as reported in the ExoCarta database (Keerthikumar et al, 2016; http://exocarta.org/exosome_markers_new). Some of these proteins such as the clathrin heavy chain, the 90 kDa heat shock protein and filamin 2 are also among the most abundant proteins found in EVs A. **Table 2** shows the 10 proteins most abundantly found in EVs of amoebae and neutrophils. The myosin heavy chain, aldehyde-alcohol dehydrogenase and 170-kDa subunit GalNAc-inhibitable lectin were the most abundant proteins in EVs A, whereas lactotransferrin, myeloperoxidase and filamin-A were the most abundant in EVs N.

**Table 1:** Typical exosomal markers found in EVs from *E. histolytica* trophozoites.

Marker (Order of abundance in 4 samples including non-annoated proteins)	Accession numbers	Exp. q-value	Sum PEP Score	Identification in 4 samples	Position in Exocharter
Clathrin heavy chain putative (12)	A0A175K294	0	281.555	4/4	23
90 kDa heat shock protein putative (28)	A0A5K1V9B6	0	154.058	4/4	10
Filamin 2 putative (33)	A0A5K1ULM5	0	133.490	4/4	42
Peroxiredoxin (46)	A0A5K1V4H1	0	107.214	4/4 4/4	31
70 kDa heat shock protein putative (48)	A0A5K1V184	0	104.387	4/4	2
Elongation factor 1-alpha (50)	A0A5K1TZM0	0	77.658	4/4 4/4	14
Glyceraldehyde-3-phosphate dehydrogenase (80)	A0A5K1VA97	0	59.190	4/4	4
Adenosylhomocysteinase (194)	A0A5K1UPJ5	0	26.791	2/4	86
Fructose 1 6-bisphosphate aldolase putative (434)	A0A5K1UI09	0	8.415	2/4	17
Profilin (439)	A0A175K0D0	0	8.292	2/4	32
Triosephosphate isomerase (542)	A0A5K1V873	0	5.886	1/4	27
Ras-related protein Rab (569)	A0A5K1VDR2	0	5.238	1/4	49
Small GTPase Rab7a (622)	A0A5K1VGP7	0	4.317	2/4	61
14-3-3 protein 3 (672)	A0A5K1VB56	0.001	3.494	3/4	15
Guanine nucleotide-binding protein subunit beta 2-l (679)	A0A5K1VNH1	0.001	3.457	3/4	38
Phosphoglycerate kinase (753)	A0A5K1UYX0	0.006	2.870	1/4	16

**Table 2:** Top 10 most abundant proteins found in EVs released by amoebic trophozoites and human neutrophils.

	Amoebic proteins	Accession number	Sum PEP Score
1	Myosin heavy chain	A0A5K1UVQ2	872.71
2	Aldehyde-alcohol dehydrogenase	A0A5K1TW74	522.68
3	Galactose-specific adhesin 170kd subunit	A0A5K1UZK4	369.85
4	Clathrin heavy chain putative	A0A175K294	281.55
5	Amylomaltase	A0A5K1UMI4	227.64
6	Proton-translocating NAD(P)(+) transhydrogenase	A0A5K1VH50	225.06
7	Calreticulin	A0A5K1V7Y1	171.06
8	90 kDa heat shock protein putative	A0A5K1V9B6	154.05
9	Filamin 2 putative	A0A5K1ULM5	133.5
10	Pyruvate ferredoxin oxidoreductase	A0A5K1VRG4	111.4
	Neutrophil proteins	
1	Lactotransferrin	P02788	806.45
2	Myeloperoxidase	P05164	525.7
3	Filamin-A	P21333	237.7
4	Moesin	P26038	154.8
5	Matrix metalloproteinase-9	P14780	116.17
6	Integrin alpha-M	P11215	110.9
7	Leukocyte elastase inhibitor	P30740	78.78
8	Catalase	P04040	72.89
9	Annexin A1	P04083	69.32
10	Alpha-enolase	P06733	64.77

Relative abundance calculated by Label-Free Quantitation (LFQ) performed in Proteome Discoverer.

## Discussion

The discovery of the ability of cells to release membranous bodies called EVs marked a before and an after in cell biology by allowing to understand how cell-to-cell communication occurs, carrying information with the ability to influence the behavior of other cells. Parasitic EVs have variable cargo depending on the developmental stage and environmental conditions, but usually carry products that can suppress the host’s innate immune response facilitating the establishment or infections (Díaz-Godínez et al., 2022).

In this work, we evaluated the size and protein content of EVs released by *E. histolytica* trophozoites alone and in co-culture with human neutrophils and determined their ability to modulate some of the effector responses of neutrophils, such as ROS production and NET release. When comparing the sizes of the EVs of amoebae and neutrophils by dynamic light scattering, we found that the variation is greater in the EVs of amoebae. The amoebae released EVs with an average size of 167 nm, but EVs of up to 600 nm were detected, which in theory could be considered as microvesicles (30 to >1000 nm, Doyle and Wang, 2019). This contrasts somewhat with the only previous study that exists on EVs of *E. histolytica* trophozoites using the same purification kit, as they report more uniform EVs of 125 nm on average (Sharma et al., 2020), suggesting variations dependent on culture conditions in each laboratory (they used 16 h culture TYI-S-33 supernatant while we used 1 h culture RPMI supernatant). On the other hand, unstimulated human neutrophils released EVs more uniform in size than those of amoeba, with an average size of 136 nm, which agrees with previous reports (Timár et al., 2013). When amoebae were co-incubated with neutrophils (1:100), it was interesting to observe the release of EVs smaller than 100 nm that had not been observed in separate cultures, and that could constitute typical exosomes. Variation not only in size, but also in cargo, is a well-known characteristic of EVs obtained under different culture conditions, stimuli, and even exposure times (Tiruvayipati et al., 2020; Kim et al., 2021). Here, we found that under coculture conditions EVs from amoebae and neutrophils tend to aggregate. We could not find information on interaction studies between EVs from different sources, and we did not carry out an additional characterization of the phenomenon, so its reason and biological significance is unknown and is the subject of current studies in our laboratory.

Human neutrophils incorporated EVs derived from amoebic trophozoites apparently by membrane fusion, as reported elsewhere in other models (Prada and Meldolesi, 2016). The red fluorescence of amoebic EVs was observed distributed in the plasma membrane of the target cell, but fluorescent dots were also observed in the cytosol suggestive of uptake of entire EVs by endocytosis. No evidence of the arrival of EVs into the nucleus was observed, but we cannot rule out the possibility that some components of their cargo could reach this organelle once released in the cytosol.

Once shown that amoeba exosomes are incorporated into neutrophils, we evaluated their effect on NETs release and coproduction of ROS, both known microbicidal mechanisms. The ability of amoebae to inhibit the respiratory burst of neutrophils in a contact-dependent manner has been reported by us and others (Arbo et al., 1990; Díaz-Godínez et al., 2021). Despite this, *E. histolytica* trophozoites are one of the most potent microbial stimuli to induce NETosis, a process that requires ROS (Díaz-Godínez and Carrero, 2019). The dilemma seems to be resolved by our previous observation that amoeba-induced NETosis depends on exogenous ROS contributed by the parasite itself (Díaz-Godínez et al., 2021). We then speculate that ROS from amoebic trophozoites might travel to the neutrophil in parasite EVs and influence neutrophil respiratory burst and NET production. The potential of EVs as carriers of ROS has been only marginally studied (Hervera et al., 2018), and to our knowledge this is the first work that demonstrates the ability of parasite EVs to transport ROS and release them into the human neutrophils. However, ROS-containing amoebic EVs did not affect the basal levels of ROS and induced very low levels of NETosis in unstimulated neutrophils, suggesting that amoeba-induced NETosis requires, in addition to parasite ROS, contact-dependent signaling mechanisms (Ávila et al, 2016; Fonseca et al, 2018).

On the contrary, the results of this work suggest that EVs rather exert a suppressive effect on NETosis induced by chemical (PMA and A23187 ionophore) and biological (the amoeba itself) stimuli. Thus, the addition of EVs from amebae or neutrophils alone (which decreased ROS production from stimulated neutrophils) or EVs from the coculture (which did not affect ROS levels), caused a delay in the onset of NETosis and in the amount of NETs released regardless of the stimulus. Together, the results suggest that EVs from quiescent neutrophils and amoebae have no effect (or little effect) on quiescent neutrophils, but instead have an immunomodulatory effect on activated neutrophils, downregulating respiratory burst and NET production. In this regard, it has been shown that the EVs of resting cells usually have suppressive effects on other cells, including ROS production and apoptosis, which contributes to the organism homeostasis (Eken et al., 2010). As examples, EVs produced by unstimulated neutrophils significantly reduced ROS and IL-8 secretion in PMA-stimulated neutrophils (Kolonics et al., 2021) and prevented the pro-inflammatory state in zymosan- and LPS-treated macrophages, reducing the expression of cytokines such as TNF-α, IL-1β, IL-6, IL-8, IL-10 or IL-12, and promoting the production of the anti-inflammatory cytokine TGF-β (Eken et al., 2010; Rhys et al., 2018). In contrast, EVs from neutrophils stimulated with zymosan-coated particles or fMLP promoted a pro-inflammatory state in unstimulated neutrophils (Kolonics et al., 2021). Interestingly, EVs from amoeba:neutrophil cocultures, which can be assumed to be from activated, or at least stimulated cells, showed a more marked inhibitory effect on NETosis. This suggests that during cell confrontation, the amoeba produces EVs with modifications in their cargo that could exert a greater immunomodulatory effect on the neutrophil, which could have important implications in the activation of immune cells and, therefore, in the host’s immune response. Certainly, we observed the appearance of smaller EVs in this condition, typical of exosomes. Another possibility is that the superior effect of the EVs of the cocultures is due to other components present in the supernatant and that copurify with the EVs, such as NETs. Although cocultures were performed for only 1 h to reduce the influence of NETs, it is possible that some released DNA and its components adhered to EVs, which could explain the aggregates we saw. The interaction of the NETs with the EVs and their effect on the immune response is a very interesting aspect to study during the interaction of the amoeba with the neutrophil, experiments that we are carrying out in our laboratory.

How EVs reduce NETosis is unknown. Since EVs from cultures alone caused a marked reduction of ROS in neutrophils, one would think that this might be the reason. However, in the case of EVs from cocultures, as mentioned above, there was no reduction of ROS in neutrophils and yet the inhibitory effect was more marked, suggesting that ROS levels might not be critical. We speculate that in the case of EVs from cocultures, in addition to the inhibitory effect due to EVs alone, the neutrophils cannot enter NETosis because they would have incorporated the EVs by phagocytosis, since as mentioned before, the EVs of cocultures tend to aggregate. This proposal is supported by a study showing that EVs from neutrophils stimulated with zymosan-coated particles or fMLP augmented phagocytosis in unstimulated neutrophils (Amjadi et al., 2021). NETosis and phagocytosis are mutually exclusive processes, since while phagocytosis requires the full functioning of the cytoskeleton, during NETosis the cytoskeleton must be dismantled (Metzler et al., 2014). Therefore, it is possible that once neutrophils have begun to phagocytize EVs from the coculture, NETosis is inhibited. Another possibility could be related with variations in the cargo of the quiescent EVs (separate cultures) vs the active ones (coculture), something that is widely reported in the literature (Shopova et al., 2020), which therefore has unpredictable effects on the target cell.

The ability of the EVs of *E. histolytica* trophozoites to transfer information between cells had not been reported until this study. In the only previous related study (Sharma et al., 2020), the EVs derived from *E. histolytica* trophozoites were characterized, but the experiment that demonstrates the transfer of information for encystment was carried out with the EVs secreted by the reptile amoeba *E. invadens*, which was used as an amoebic encystment model due to the difficulty in encysting *E. histolytica in vitro* (Aguilar-Díaz et al., 2011). Here we show that EVs secreted by *E. histolytica* trophozoites can be incorporated into human neutrophils transferring their cargo (including ROS), affecting respiratory burst and NETosis. To identify proteins that could participate on the effect of EVs on neutrophils, we determined the proteome of EVs from amebae and neutrophils in separate cultures and compare them against the proteome of EVs derived from cocultures. For the purification of amoebic EVs, we used the same exosome isolation kit previously used by Sharma et al. (2020), because they had successfully obtained EVs and because we wanted to make a comparison with their proteome results. We also use the same method for the purification of EVs from neutrophils and co-cultures for comparison purposes. Regarding the number of different proteins, we identified almost 600, which is close to the 700 identified by them. The Gene Ontology analysis returned results that agree with theirs in that most of the proteins are grouped within the categories of catalytic activity and binding, which reinforces the presence of proteins with these activities in the EVs secreted by *E. histolytica* trophozoites. Likewise, the proteome of this work shows the conclusive identification in EVs of amoebae of 16 orthologs of the top 100 of the exosome markers most frequently identified in mammals and integrated in Exocarta. As in Sharma et al. (2020), we repeatedly identified heat shock protein 70 and the elongation factor 1-alpha but did not find ADP-rybosylation factor. Although HSP70 is a stress-associated protein, it is reported in the Exocarta list as the second most frequently found in studies. Instead, we identified clathrin heavy chain, followed by HSP90, filamin, peroxiredoxin, and glyceraldehyde-3-phosphate dehydrogenase, as the most abundant typical exosome proteins present in all processed amoebic EVs samples. The fact that amoeba EVs contain some of the typical exosome markers, but lacks tetraspanins, the main exosome marker in mammals and of which amoeba has 17 potentially encoding genes (Tomii et al, 2019), supports the proposal of Sharma et al. (2020) that the biogenesis and secretion of EVs in amoeba is a partially conserved process.

Apart from exosomal marker orthologs, we identified the GalNAc-inhibitable lectin and calreticulin as two of the most abundant proteins in *E. histolytica* EVs. Even more interesting was finding both proteins as those that most increased their relative abundance in the EVs of the cocultures. Both proteins bind carbohydrates, and among their many functions, they have been implicated in cell signaling processes and control of gene expression, so they are candidates to be responsible, at least in part, for some of the effects reported here of amoebic EVs on human neutrophils (Petri et al., 2002; Kammanadiminti et al., 2004). The transfer of the lectin to target cells in the host within the first 5 minutes of incubation has been previously reported (Leroy et al., 1995), which could be occurring through the EVs. On the other hand, the induction of signaling by Gal-lectin through TLRs 2 and 4 has been reported in human colonic cells and in macrophages -activating the inflammasome in the latter-, so this amoebic molecule can be considered as a genuine PAMP and signaling promoter (Kammanadiminti et al., 2004; Galván-Moroyoqui et al., 2011; Mortimer et al., 2014). It has recently been reported that Gal-lectin can also act as a ligand for the c-Met receptor on the surface of HepG2 cells, and that its occupancy by its natural ligand hepatocyte growth factor, prevents cytotoxic damage to liver cells due to the amoeba, without affecting its adherence (Pérez-Hernández et al., 2021). Regarding *E. histolytica* calreticulin, this protein has been found in cytoplasmic vesicles of different sizes (which is in accordance with its presence in the EVs reported here) as well as on the surface of the parasite, and it is relocated in the phagocytic cups when the amoeba ingests red blood cells, in addition to transferring and joining apoptotic lymphocytes and human C1q (González et al., 2011; Vaithilingam et al, 2012). The presence of calreticulin in EVs suggest that the transfer process could be occurring through them. In addition, amoebic calreticulin has mitogenic activity and acts as an immunogen for the activation of peripheral blood mononuclear cells from patients with amoebic liver abscesses (Gonzalez-Rivas et al., 2018), suggesting that it can signal and induce changes in the expression of target cells. However, the most abundant proteins found in the EVs from *E. histolytica* by far were myosin heavy chain and aldehyde-alcohol dehydrogenase, proteins that have been associated, together with Gal-lectin, in the formation of the uroid region during cappin, folding process of the membrane towards the back of the parasite by which the amoeba releases caps to the extracellular media removing antibodies and any opsonizing molecule from its surface (Rahim et al., 1993; Arhets et al., 1995). The abundant of these proteins in the EVs of the amoeba could have to do with their ubiquitous cellular location, since they have been described both in the cytosol and in the membrane of the parasite. This may also indicate an association between the formation of surface structures such as the uroid region and the release of EVs, something that warrants further study. Finally, despite the large number of proteins with multiple functions found in the EVs of amoebae that could be responsible for the immunomodulatory effect seen on respiratory burst and NET formation in neutrophils, we cannot rule out the participation of other components of cargo, particularly the previously described multiple RNAs (Sharma et al., 2020).

Overall, the results of this work together with our previous findings demonstrate that the interaction of the amoeba with the neutrophil is a complex interplay, in which the amoeba usually wins as it has also been suggested elsewhere (Chadee and Meerovitch, 1984; Tsutsumi et al., 1984; Salata and Ravdin., 1986). In the encounter, both the amoeba and the neutrophil release EVs, of which at least the EVs of the amoebae, as we showed here, are incorporated into the neutrophils, affecting their effector functions, probably at a distance. Our LFQ data suggest that during confrontation, both cells respond by increasing the abundance of key proteins for attack and/or manipulation of the opposite cell. Thus, the neutrophil reacts by releasing EVs with a higher content of key antimicrobial molecules, such as lactotransferrin, myeloperoxidase, elastase and defensins, which will invariably try to kill the amoeba. The potent anti-amoebic effect of lactotransferrin (lactoferrin) and derived peptides was previously reported by us, not only killing *E. histolytica* trophozoites *in vitro*, but also resolving amoebic intracecal infection in mice (León-Sicairos et al., 2012; Díaz-Godínez et al., 2019). *In vitro* killing of amoeba by myeloperoxidase has also been reported (Pachecho-Yepez et al., 2011). However, under contact, amoebae activate NETosis (a process in which ROS from amoebae transported in EVs seem to participate; Díaz-Godínez et al., 2021) destroying the neutrophil, which may contribute to tissue damage due to the cytolytic effect of the associated microbicidal components and due to the inflammation that NETs promote by activating complement and coagulation (de Bont et al., 2019). These proposals merit additional studies that are being carried out in our laboratory and that will help to understand the role of neutrophils in the pathogenesis or protection against invasive amebiasis.

## Data availability statement

The datasets presented in this study can be found in online repositories. The names of the repository/repositories and accession number(s) can be found below: The mass spectrometry proteomics data have been deposited to the ProteomeXchange Consortium *via* the PRIDE [1] partner repository with the dataset identifier PXD036228 and 10.6019/PXD036228.

## Ethics statement

This study was carried out in accordance with the recommendations and approval of the Ethical Committee for Studies on Humans of the Instituto de Investigaciones Biomédicas, UNAM (Ethical approved number: FMED/CI/RGG/013/01/2018). All subjects signed a written informed consent.

## Author contributions

Conceptualization, JC, SM-C and CD-G. Methodology, CD-G, DR-V and SG-A. Validation, CD-G and SG-A. Formal analysis, JC, SM-C and CD-G. Investigation, CD-G and JC. Resources, JC. Data curation, JC, SM-C and CD-G. Statistical analysis, CD-G and DR-V. Writing, CD-G and JC. Supervision, JC and SM-C. Project administration, JC. Funding acquisition, JC. All authors contributed to the article and approved the submitted version.

## Funding

This research was funded by Consejo Nacional de Ciencia y Tecnología (CONACyT), grant number 284830 (to JC) and Programa de Apoyo a Proyectos de Investigación e Innovación Tecnológica (PAPIIT-UNAM), grant number IN208020 (to JC).

## Acknowledgments

SG-A is a student of the Programa de Ciencias Biológicas, UNAM, and is a recipient of a scholarship from Consejo Nacional de Ciencia y Tecnología (CONACyT), Mexico (1174212).

## Conflict of interest

The authors declare that the research was conducted in the absence of any commercial or financial relationships that could be construed as a potential conflict of interest.

## Publisher’s note

All claims expressed in this article are solely those of the authors and do not necessarily represent those of their affiliated organizations, or those of the publisher, the editors and the reviewers. Any product that may be evaluated in this article, or claim that may be made by its manufacturer, is not guaranteed or endorsed by the publisher.
