# Supplementary material for: Immunomodulatory effect of extracellular vesicles from Entamoeba histolytica trophozoites: Regulation of NETs and respiratory burst during confrontation with human neutrophils
Source: Front Cell Infect Microbiol. 2022 Oct 28;12:1018314. doi: 10.3389/fcimb.2022.1018314 (PMC9650183; doi:10.3389/fcimb.2022.1018314)
Supplement: Supplementary file 1 [file DataSheet_1.zip › Supplementary Tables 1 to 4.docx]

**Supplementary Table 1. Amoebic proteins identified in EVs uniquely during trophozoite-neutrophil interaction.**

|  | Protein | Accesion number | Putative Function |
| --- | --- | --- | --- |
| 1 | Alpha-1,4 glucan phosphorylase | A0A175JRF8 | Glycogen catabolism |
| 2 | Aminopeptidase putative | A0A5K1VFQ4 | Proteolysis |
| 3 | Bromodomain-containing protein | A0A5K1UVP7 | Epigenetic reader |
| 4 | Inositol polyphosphate 5-phosphatase putative | A0A5K1V3I8 | Cellular signaling |
| 5 | Lipid phosphate phosphatase putative | A0A5K1VHX7 | Cellular signaling |
| 6 | Malate dehydrogenase | A0A5K1VES0 | Malate metabolic process |
| 7 | p-glycoprotein 6 putative | A0A175JTA3 | ABC-type transmembrane transporter |
| 8 | Thioredoxin putative | A0A5K1V168 | Antioxidant system |
| 9 | UDP-N-acetylglucosamine transporter putative | A0A5K1TUJ6 | Transports across the Golgi membrane |
| 10 | Uncharacterized protein * | A0A175JK68 | Unknown |
| 11 | Uncharacterized protein * | A0A5K1TV08 | Unknown |
| 12 | Uncharacterized protein * | A0A5K1VIJ5 | Unknown |
| 13 | Uncharacterized protein | A0A5K1U9U4 | Unknown |
| 14 | Uncharacterized protein | A0A175JWC9 | Unknown |
| 15 | Uncharacterized protein | A0A175JK32 | Unknown |
| 16 | Uncharacterized protein | A0A5K1V5K9 | Unknown |

***** The sequences of these proteins showed 100% match in BLASTp with a sequence of *Entamoeba histolytica* HM-3:IMSS, described as a putative T-cell immunomodulatory protein precursor (EMS15946.1).

**Supplementary Table 2. Main amoebic proteins overexpressed in EVs from *E. histolytica* trophozoites in coculture with human neutrophils.**

|  | Protein | Accession number | Sum PEP Score | Fold change *  S1, S2, S3, S4 |
| --- | --- | --- | --- | --- |
| 1 | **Galactose-inhibitable lectin 170 kDa subunit** | **A0A5K1VUZ3** | **369.854** | **5.7, 6.2, 4.4, 3.8** |
| 2 | **Calreticulin** | **A0A5K1V7Y1** | **171.064** | **2.7, 2.0, 1.5, 1.6** |
| 3 | **Peroxiredoxin** | **A0A5K1V4H1** | **107.214** | **5.3, 3.9, 4.0, 7.0** |
| 4 | 70 kDa heat shock protein putative | A0A5K1V184 | 104.387 | 4.0, 5.4, ND, 2.0 |
| 5 | Actin binding protein putative | A0A5K1UVA9 | 101.124 | 4.4, 4.0, 12.7, 8.0 |
| 6 | Alpha-amylase family protein | A0A5K1V199 | 73.017 | 4.4, 6.5, 5.1, 6.7 |
| 7 | Dipeptidyl-peptidase putative | A0A175JGD5 | 71.136 | 6.5, 5.8, 11.6, 14 |
| 8 | Serine carboxypeptidase s28 family protein | A0A5K1TXZ9 | 20.641 | ND, ND, 8.0, 8.8 |

* Relative abundance calculated by spectral count with normalization set to total peptide amount in each sample. Gene ontology analysis was performed using PANTHER GO platform.

S: sample

ND: No detected in the corresponding sample.

Bold: Increase clearly detected in the four samples of coculture processed.

**Supplementary Table 3. Neutrophil antimicrobial proteins and peptides detected in EVs uniquely during neutrophil-trophozoite interaction.**

|  | Protein | Accession number | Function | Reference |
| --- | --- | --- | --- | --- |
| 1 | Bactericidal permeability-increasing protein | P17213 | LPS neutralization, chemoattractant, opsonization | Balakrishnan et al., 2012 |
| 2 | Cathelicidin antimicrobial peptide | P49913 | Antimicrobial peptide | Kościuczuk et al., 2012 |
| 3 | Dermicidin | P81605 | Antimicrobial peptide | Schittek et al., 2001 |
| 4 | Histone H2B type 2-E | Q16778 | Antimicrobial effect | Kawasaki et al., 2008 |
| 5 | Histone H3.3 | P84243 | Antimicrobial effect | Hoeksema et al., 2016 |
| 6 | Neutrophil defensin | P12838 | Antimicrobial peptide | Chaly et al., 2000 |
| 7 | Serine protease 57 | A0A0A0MR61 | Antimicrobial effect, degrades virulence factors | Pham, 2006 |

**Supplementary Table 4.** **Main human neutrophil proteins overexpressed in EVs from neutrophils in coculture with *E. histolytica* trophozoites.**

|  | Protein | Accession number | Sum PEP Score | Fold change*  S1, S2, S3, S4 |
| --- | --- | --- | --- | --- |
| 1 | **Lactotransferrin** | **P02788** | **806.45** | **2.5, 2.6, 4.1, 4.0** |
| 2 | **Myeloperoxidase** | **P05164** | **525.68** | **3.4, 3.5, 3.4, 5.0** |
| 3 | Neutrophil elastase | P08246 | 51.641 | 1.4, 0.0, 2.5, 2.0 |
| 4 | Eosinophil peroxidase | P11678 | 39.805 | ND, ND, 3.7, 4.6 |
| 5 | Cathepsin G | P08311 | 32.554 | 5.2, 2.8, 4.5, 3.7 |
| 6 | Calreticulin | P27797 | 20.732 | ND, ND, 4.6, 1.4 |
| 11 | Neutrophil defensin 1 | P59665 | 11.631 | 2.0, 3.0, 0.0, 2.0 |

* Relative abundance calculated by spectral count with normalization set to total peptide amount in each sample. Gene ontology analysis was performed using PANTHER GO platform.

S: sample

ND: No detected in the corresponding sample.

Bold: Increase clearly detected in the four samples of coculture processed.
